# Short-term benefits of adaptive sporting events on social and leisure satisfaction in veterans with disabilities: impact of military service era and medical diagnosis

**DOI:** 10.3389/fspor.2026.1773675

**Published:** 2026-06-19

**Authors:** Alexis N. Sidiropoulos, Jonathan J. Glasberg, David V. Herlihy, John M. Chomack, Timothy E. Moore, Eric Bae, Leif M. Nelson, Jason T. Maikos

**Affiliations:** 1 Prosthetics and Sensory Aids Service, Department of Veterans Affairs, New York Harbor Healthcare System, New York, NY, United States; 2 Narrows Institute for Biomedical Research and Education, Inc., Brooklyn, NY, United States; 3 Statistical Consulting Services, Center for Open Research Resources and Equipment, University of Connecticut, Storrs, CT, United States; 4 National Veterans Sports Programs and Special Events, Department of Veterans Affairs, Washington, DC, United States

**Keywords:** adaptive sports, medical diagnosis, military service era, quality of life, social and leisure participation, veterans

## Abstract

**Introduction:**

Veterans with physical or mental health disabilities often experience reduced quality of life following military service. Adaptive sporting events may help mitigate these challenges by providing physical, social, and psychological benefits. This study examined the acute effects of single-day adaptive sporting events on satisfaction with social and leisure participation in veterans with disabilities. We hypothesized that participation would acutely improve quality-of-life-related outcomes.

**Methods:**

Participants engaged in either an adaptive kayaking and sailing event or an adaptive cycling and hiking event and completed the PROMIS Satisfaction with Participation in Discretionary Social Activities survey before and after the event. Mixed-effects models were used to estimate predicted T-scores, adjusting for military service era, medical diagnosis, sex, and event type.

**Results:**

Findings indicated that participation in adaptive sporting events was associated with modest but statistically significant increases in predicted T-scores following the events. Additionally, veterans from more recent service eras reported lower predicted quality-of-life-related T-scores at baseline, whereas veterans with limb loss had higher predicted T-scores than those with other diagnoses. Improvements were most apparent among veterans with lower or intermediate baseline satisfaction, whereas ceiling effects limited observable change among those with higher baseline scores.

**Discussion:**

These results suggest that adaptive sports can provide immediate psychosocial benefits, and may be particularly valuable for veterans with lower baseline satisfaction with social and leisure participation. Future adaptive sporting events should prioritize engagement of veterans from recent conflicts to optimize short-term improvements in quality of life. These findings highlight the value of adaptive sports as a low-barrier, community-based intervention for enhancing social and leisure satisfaction in veterans living with disabilities.

## 1 Introduction

Veterans with disabilities often report lower quality of life (QoL) than civilians living with disabilities, in part due to physical and psychological consequences of military service (1). Although regular physical activity is strongly associated with improved QoL through well-established physical and psychosocial benefits (2, 3), many veterans with physical and mental disabilities face a complex array of interconnected barriers to physical activity including chronic pain, musculoskeletal disorders, arthritis, and cardiopulmonary disease (4). Functional challenges like activity limitations and equipment issues, including poorly fitting prostheses, also significantly impede participation (5). These physical obstacles are compounded by prominent mental and psychological barriers including lack of energy and motivation (6), alongside critical confidence-related problems such as lower physical activity self-efficacy (7) and a self-perceived lack of capability (4). These barriers can limit participation and sustained involvement in traditional forms of physical activity, preventing veterans from fully realizing the QoL benefits typically associated with an active lifestyle.

Adaptive sports provide an inclusive, participation-focused approach to physical activity by modifying equipment, environments, and rules to meet the needs of individuals with disabilities (8, 9). Participation in adaptive sports has been associated with improvements across multiple health domains, including physical function, psychological well-being, and social connectedness (10–14). These outcomes align closely with key dimensions of QoL and support continued investigation into the impact of adaptive sports as a means of promoting active living among veterans with disabilities. Veterans represent a heterogeneous population, with differences in military service era reflecting variation in injury mechanisms, rehabilitation exposure, psychosocial context, and expectations surrounding physical activity and active living, all of which may influence baseline QoL and responsiveness to adaptive sport participation.

Veterans also present with a wide range of service- and non-service-related health conditions, including limb loss, sensory impairments (i.e., visual and hearing impairments), musculoskeletal disorders, neurologic disorders, and mental health conditions. Social factors, including a lack of social support (4) and unsupportive others (5), further complicate engagement. Despite these substantial challenges, many inactive veterans report an interest in initiating exercise, with a preference for structured, supervised, and socially supportive environments (6). Differences in functional capacity, access to assistive technologies, environmental barriers, and prior rehabilitation experiences associated with these diagnoses may further influence QoL-related outcomes and responses to adaptive sports participation (15, 16). Examining outcomes across medical diagnoses can therefore help clarify which veterans may benefit most acutely from participation and inform the design of inclusive, targeted adaptive sport programming.

In addition to long-term engagement, adaptive sporting events may exert meaningful short-term effects on QoL through immediate social interaction, perceived competence, and participation in supportive, non-clinical environments (17, 18). Examining acute changes in satisfaction with social and leisure participation following community-based adaptive sporting events provides insight into short-term QoL-related responses, and how these responses vary across veterans with differing military service eras and medical diagnoses. Previous literature supports this work, as participating in a brief, day-long outdoor recreational activity intervention was associated with a variety of improved mental health symptoms (19). Our previous work demonstrated an acute, positive effect of adaptive sport participation on QoL-related outcomes in veterans with disabilities (17). However, that study was limited by a smaller sample size and non-standardized outcome measures that did not allow for detailed subgroup analysis. The present study builds on this prior work by including a larger cohort, enabling subgroup comparisons based on military service era and medical diagnosis, and using a validated patient-reported outcome measure. Specifically, the Patient-Reported Outcomes Measurement Information System (PROMIS) Satisfaction with Participation in Discretionary Social Activities measure was selected because it captures satisfaction with social and leisure participation, which are domains of QoL that are particularly responsive to short-term changes in social context, perceived inclusion, and engagement in a meaningful activity (20). Understanding acute QoL-related responses has direct translational relevance for the design and implementation of community-based adaptive sport programs that support active living beyond the clinical setting.

The purpose of this study was to examine the acute effects of participation in single-day adaptive sporting events on QoL-related outcomes in veterans living with disabilities and to evaluate whether responses differed across key subgroups defined by military service era and medical diagnosis. The primary outcome was PROMIS Satisfaction with Participation in Discretionary Social Activities T-scores assessed prior to the events and immediately after participation. It was hypothesized that participation in a single-day adaptive sporting event would, on average, be associated with statistically significant increases in PROMIS T-scores from pre- to post-event assessment, reflecting acute improvements in satisfaction with social and leisure participation. We further hypothesized that the magnitude of pre- to post-event change would differ across military service era and across medical diagnosis subgroups, defined as significant time × era and time × diagnosis interaction effects in mixed-effects models.

## 2 Methods

### 2.1 Participants

This study included data from veterans who participated in one-day community-based adaptive sporting events between 2022 and 2025. Two annual adaptive sporting events were included: a kayaking and sailing event and a cycling and hiking event. All veterans who registered for and participated in the events were eligible for inclusion. Medical diagnoses were identified through retrospective review of electronic medical records. Diagnoses were then broadly grouped into the following categories: limb loss (i.e., upper and/or lower extremity), sensory impairment (i.e., visual and hearing), musculoskeletal conditions (i.e., chronic orthopedic conditions and Multiple Sclerosis), mental health diagnoses (i.e., Post-Traumatic Stress Disorder, Depression, Schizophrenia, Anxiety Disorder), and neurologic conditions (i.e., Spinal Cord Injury, Stroke, Traumatic Brain Injury, Amyotrophic Lateral Sclerosis, Parkinson's Disease). When veterans had multiple documented diagnoses, classification was based on the condition judged to be most functionally limiting with respect to physical activity participation, consistent with prior adaptive sport program evaluation approaches. This grouping strategy was used to facilitate interpretable subgroup analyses while acknowledging clinical heterogeneity within categories.

Military service era was categorized *a priori* to reflect differences in injury mechanisms, rehabilitation exposure, and psychosocial context. Veterans were grouped into five service eras: Vietnam, Post-Vietnam, Gulf War, Post-Gulf War, and Operation Enduring Freedom/Operation Iraqi Freedom (OEF/OIF). These groupings were selected to align with prior literature and to support planned subgroup analyses examining whether baseline QoL-related outcomes and acute responses to adaptive sport participation differed across cohorts with distinct service-related experiences.

This study involved a retrospective analysis of prospectively collected program evaluation data and was approved by the Veterans Affairs New York Harbor Healthcare System Institutional Review Board (Protocol #1628), which granted a waiver of documentation of informed consent due to the retrospective nature of the analysis. All participants received medical clearance from their primary care or rehabilitation specialty provider prior to participation in the adaptive sporting events.

### 2.2 Adaptive sporting events

The adaptive kayaking and sailing event has been described previously (17). In brief, veterans received professional instruction in sailing and kayaking through VA community partners and participated in approximately two hours of each activity. The adaptive cycling and hiking event consisted of a full-day instructional clinic designed to teach veterans with disabilities how to participate in adaptive cycling and hiking using modified equipment. Both events were conducted annually and designed to promote physical activity, social interaction, and participation in sport outside of the typical clinical rehabilitation setting for veterans living with disabilities in the New York and New Jersey region.

### 2.3 Outcome measures

Demographic data collected included age, sex, military service era, and disability diagnosis. QoL-related outcomes were assessed using the PROMIS Short Form-7a assessing Satisfaction with Participation in Discretionary Social Activities (Supplementary Figure A). This measure is a valid and reliable patient-reported outcome measure of satisfaction with social and leisure participation that reflects QoL-related social health, including satisfaction with leisure and social participation (21). PROMIS measures are widely used in both clinical and research settings and are designed to be sensitive to change across a range of health conditions (22, 23). Previous research supports use of this tool in both 7-day and no recall conditions (24, 25). Responses were recorded on a 5-point Likert scale and converted to standardized T-scores, ranging from 20 to 80, with higher scores indicating greater satisfaction. Surveys were administered in hard-copy format 2–4 weeks prior to participation and again immediately following completion of the adaptive sporting event. This timing was selected to capture acute changes associated with participation while minimizing respondent burden.

### 2.4 Statistical analysis

The aim of statistical analyses was to evaluate the effects of military service era (5 levels: Vietnam, Post-Vietnam, Gulf, Post-Gulf, OEF/OIF) and medical diagnoses (5 levels: Limb Loss, Mental Health, Sensory Impairment, Musculoskeletal Impairments, Neurologic Impairments) on changes in PROMIS T-scores before and after participation in community-based adaptive sporting events. Event type (kayaking and sailing vs. cycling and hiking) and sex were included as covariates.

To account for the bounded nature of PROMIS T-scores and the presence of ceiling effects (values at the upper bound) at both pre- and post-event assessments, Bayesian mixed-effects zero-one inflated beta (ZOIB) models were used. This modeling approach allows simultaneous estimation of non-maximum T-scores using fixed effects while also estimating the probability of attaining the maximum T-score. T-scores were rescaled by dividing by the maximum observed value to constrain responses to the (0,1) interval. Fixed effects included time (pre-event vs. post-event), military service era, event type, sex, and medical diagnosis (see Supplementary Table A for details). Interaction terms between time and diagnosis were included to assess whether pre- to post-event changes differed across diagnosis subgroups. Participant identification was included as a random effect to account for repeated measures within individuals.

Model-based (marginal) predicted T-scores were derived to estimate adjusted mean PROMIS T-scores for specified groups while holding other covariates constant. These predicted values were used for visualization and subgroup comparisons. Because the analyses were conducted using Bayesian inference, traditional frequentist *p*-values were not reported. Instead, statistical significance was evaluated using 95% Credible Intervals (CrIs) on the logit scale of the predicted T-scores, with effects considered statistically significant when the interval did not include zero (i.e., the weight of evidence supports a non-zero effect).

## 3 Results

Data from 124 veterans who participated in single-day adaptive sporting events between 2022 and 2025 were initially available for analysis. Four participants were excluded due to missing diagnostic information, resulting in an analytic sample of 120 veterans and 452 pre- and post-event observations, as some veterans participated in multiple events (Table 1). Most participants were male, from the Post-Vietnam military service era, and diagnosed with a neurologic impairment. Participation was higher in kayaking and sailing events than in cycling and hiking events.

**Table 1:** Demographic characteristics of participants (*n* = 124).

Characteristics	Number of Participants
Military Service Era	
Vietnam	35 (28.2%)
Post-Vietnam	48 (38.7%)
Gulf	11 (8.9%)
Post-Gulf	10 (8.1%)
OEF/OIF	20 (16.1%)
Diagnosis	
Mental Health	30 (24.2%)
Limb Loss	10 (8.1%)
Sensory Impairment	22 (17.7%)
Musculoskeletal Impairment	20 (16.1%)
Neurologic Impairment	38 (30.6%)
Unknown	4 (3.2%)
Sex	
Male	96 (77.4%)
Female	28 (22.6%)
Age	
Mean (Standard Deviation)	63 (12)
Median (Min, Max)	63 (19, 85)
Event	
Kayaking/Sailing	68 (54.8%)
Cycling/Hiking	26 (21.0%)
Both	30 (24.2%)

### 3.1 Overall acute change in PROMIS T-scores

After adjusting for military service era, medical diagnosis, event type, sex, and participant-level random effects, participation in adaptive sporting events was associated with a modest but statistically significant increase in post-event predicted PROMIS T-scores among participants whose pre-event scores were not at the maximum (95% CrI 0.02–0.44; Supplementary Table B). These improvements were most apparent among veterans with lower or intermediate baseline T-scores. Ceiling effects limited detectable change among veterans with high baseline scores; however, participation in adaptive sporting events was also associated with a significant increase in the probability of attaining maximum predicted PROMIS T-scores (95% CrI: 0.30–1.06; Supplementary Table C).

### 3.2 Event type

Event type was a significant predictor of PROMIS T-scores, with veterans participating in the kayaking and sailing events demonstrating higher predicted T-scores at both pre- and post-event assessments compared with those participating in the cycling and hiking event (95% CrI: 0.34–2.49; Supplementary Tables B, D). However, no significant interaction between event type and time was observed, indicating that differences between events reflected baseline differences rather than differential responsiveness to participation.

### 3.3 Military service era

**Figure 1:**
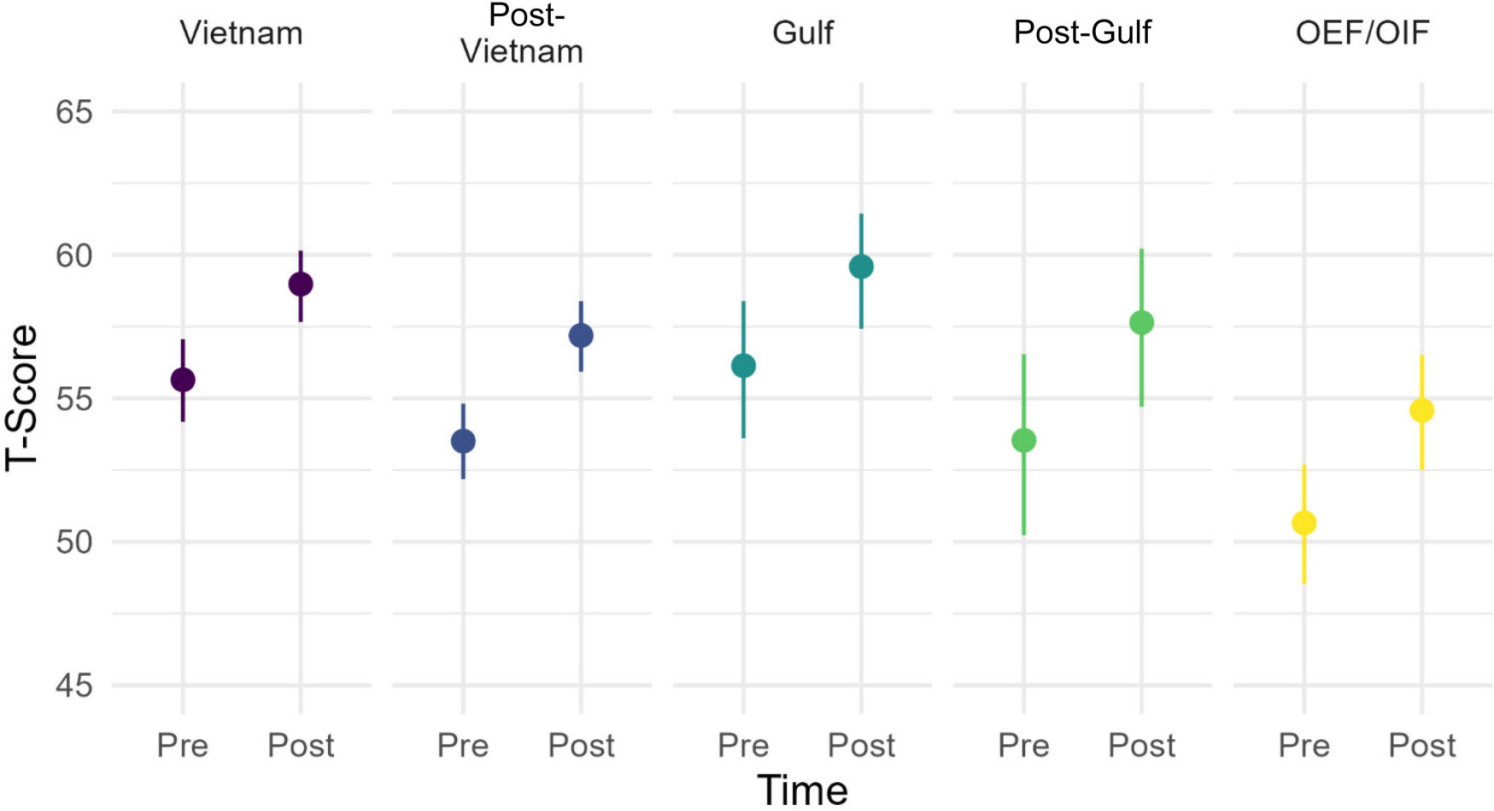
Pre- and post-event T-score values by military service era. OEF, Operation Enduring Freedom; OIF, Operation Iraqi Freedom.

After adjusting for event type, sex, medical diagnosis, and participant-level random effects, predicted PROMIS T-scores differed significantly across military service eras (Supplementary Table B). *Post hoc* contrasts indicated that veterans from more recent service eras generally reported lower predicted T-scores compared with those from earlier eras (Figure 1; Supplementary Table D). Specifically, OEF/OIF veterans demonstrated significantly lower predicted T-scores than Vietnam-era veterans (95% CrI: −7.44–−1.96), Gulf-era veterans (95% CrI: −8.55–−1.84), and Post-Vietnam veterans (95% CrI: −5.55–−0.01). In contrast, differences among Gulf, Post-Vietnam, and Post-Gulf eras were otherwise not statistically significant (95% CrI included 0), indicating that the largest differences were observed between the earliest and most recent service eras. Predicted pre- and post-event scores by military service era are presented in Figure 1 and Supplementary Table E.

### 3.4 Medical diagnosis

**Figure 2:**
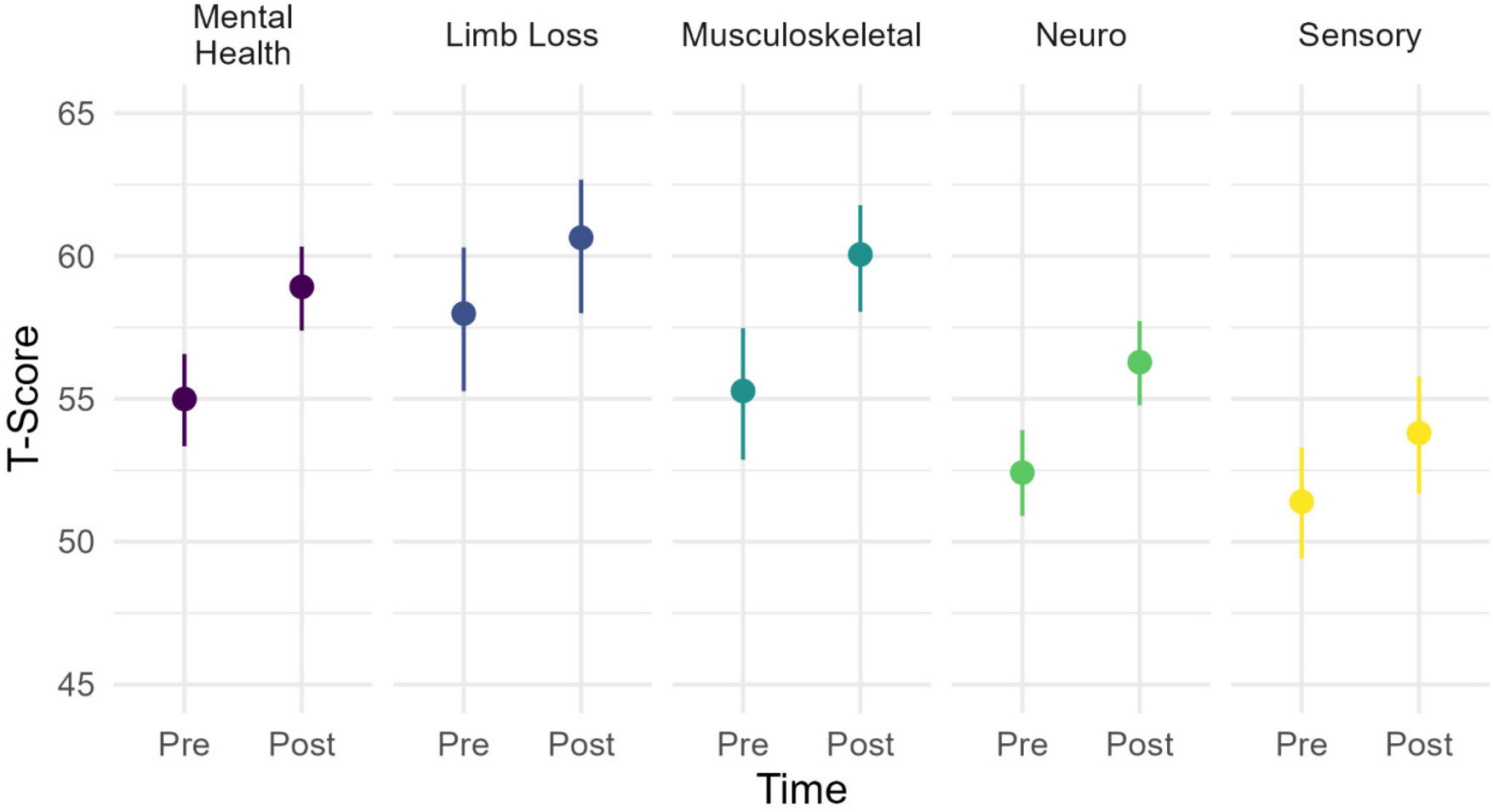
Pre- and post-event T-score values by medical diagnosis.

Diagnosis category was not a significant overall predictor of PROMIS T-scores after adjusting for military service era, event type, and sex (Supplementary Table B). However, *post hoc* contrasts of marginal predicted T-scores revealed significant differences between specific diagnostic groups (Figure 2, Supplementary Table D). Veterans with limb loss demonstrated higher predicted T-scores compared with those with sensory impairments (95% CrI: 3.32–9.80) and neurologic impairments (95% CrI: 1.66–7.79). Predicted T-scores did not differ significantly between veterans with limb loss and those with mental health diagnoses and musculoskeletal conditions. Veterans with sensory impairments also exhibited lower predicted T-scores compared with those with mental health diagnoses (95% CrI: −6.97–−1.75) and musculoskeletal conditions (95% CrI: −8.10–−1.90). No significant interactions between time and diagnosis category were observed, indicating that the magnitude of pre- to post-event change did not differ significantly across diagnosis groups. Veterans with limb loss demonstrated minimal change over time, consistent with higher baseline satisfaction and potential ceiling effects. Predicted pre- and post-event T-scores by diagnosis category are presented in Figure 2 and Supplementary Table E.

## 4 Discussion

The purpose of this study was to examine the acute effects of participation in a single-day adaptive sporting event on satisfaction with social and leisure participation among veterans living with disabilities, with planned subgroup analyses by military service era and medical diagnosis. Consistent with the study hypothesis and our prior work, participation in adaptive sporting events was associated with modest, but statistically significant acute improvements in predicted PROMIS T-scores, reflecting short-term improvements in satisfaction with social and leisure participation. These improvements were most apparent among veterans with lower or intermediate baseline scores, while ceiling effects limited observable change among those with high baseline scores. These findings support community-based adaptive sports events as a meaningful form of non-traditional rehabilitation that may promote psychosocial well-being and active living among veterans living with disabilities. However, short-term gains likely arise from multiple interacting factors, including physical activity, novelty, social support, and temporary disruption of daily routines, which are difficult to disentangle in a single-day design.

The absence of differences in pre- to post-event change between adaptive sporting events suggests that acute improvements in QoL-related outcomes were not specific to the type of sport and is consistent with previous literature (14). Rather, adaptive sports in general may confer acute benefits through shared elements, such as physical activity, social interaction, peer support, community engagement, and opportunities for mastery and enjoyment. Conclusions generated from previous literature support this notion, stating that providing therapeutic outdoor recreation activities that are novel and introduce veterans to new peers may be more successful at both recruitment and retention than traditional treatment approaches (26). Future research should explicitly explore the influence of non-physical components on QoL-related outcomes.

### 4.1 Military service era

Lower predicted PROMIS T-scores were observed among veterans who served in more recent military conflicts, particularly those from the OEF/OIF era, compared with veterans from earlier service eras. These differences may reflect the higher prevalence of post-deployment mental and physical health conditions among the more recent cohort, including post-traumatic stress disorder, depression, traumatic brain injury, and complex polytrauma, which can negatively influence satisfaction with social and leisure participation (10, 27). Broader psychosocial challenges in this population, including social isolation, diminished sense of purpose, and reduced psychological well-being (28), may also contribute.

Differences across service eras may also reflect variation in expectations for physical activity and lifestyle. Veterans from OEF/OIF may have higher expectations for maintaining an active lifestyle (29, 30), such that disability-related limitations represent a greater perceived disruption to anticipated levels of activity and independence. This expectation mismatch may negatively influence satisfaction-based measures such as the PROMIS instrument used in this study. In contrast, veterans from earlier service eras may have recalibrated expectations over time, contributing to higher baseline satisfaction despite age-related functional changes.

Observed differences may further reflect variation in life stage and role demands rather than age alone. Veterans from earlier service eras may have fewer competing work and caregiving responsibilities due to being in or near retirement age, allowing for greater flexibility in leisure and social participation. Because the PROMIS instrument in this study assessed satisfaction with participation rather than objective opportunity or physical capacity, life context may meaningfully shape reported outcomes. Time since injury and psychosocial adaptation likely play an important role as well, with veterans from earlier service eras having more time to integrate disability into their identities, develop compensatory strategies, and establish stable social and leisure routines. In contrast, veterans from more recent conflicts may be earlier in this adjustment process, with ongoing identity reconstruction and evolving participation patterns (31, 32). Because PROMIS domains assessed in this study emphasize satisfaction with leisure and social roles, these measures may be particularly sensitive to long-term adaptation and fulfillment of expectations rather than physical ability alone.

### 4.2 Medical diagnosis

Differences in QoL-related outcomes were also observed across medical diagnosis subgroups. Veterans with limb loss demonstrated higher predicted PROMIS T-scores compared with veterans with sensory and neurologic impairments, indicating consistently greater satisfaction with social and leisure participation in this subgroup.

Lower satisfaction among veterans with sensory impairments is consistent with prior research demonstrating that individuals with visual and hearing impairments frequently report reduced QoL compared with other disability groups, particularly in domains related to social participation and environmental accessibility (33, 34). Systematic reviews have identified significant associations between both visual and hearing impairments and poorer overall QoL, with more pronounced effects observed among individuals with dual sensory loss (33). Population-based studies have similarly shown that sensory impairment negatively affects social functioning and participation, contributing to reduced satisfaction with community engagement (34). Psychosocial mechanisms, including increased risk of loneliness, smaller social networks, and depressive symptoms, have been identified as partial mediators of the relationship between sensory loss and diminished QoL (35). Together, these findings suggest that lower baseline satisfaction in veterans with sensory impairments may reflect well-documented participation and accessibility barriers that extend beyond the context of a single adaptive sporting event. In addition to these broader participation challenges, the sensory and environmental demands of the adaptive sporting events may have influenced subjective satisfaction. These activities relied heavily on visual or auditory input within dynamic outdoor settings, and instructors may not regularly work with individuals with sensory challenges. Our findings align with this broader literature and may reflect both pre-existing participation constraints and contextual demands specific to the adaptive sporting events evaluated in the present study. These findings should not be interpreted as a diminished benefit of adaptive sports for veterans with sensory impairments, but rather these contextual factors may represent a potential mismatch between impairment-specific needs and the demands of the selected activities.

Notably, veterans with limb loss demonstrated minimal observable change in predicted PROMIS T-scores, which may reflect higher baseline satisfaction and potential ceiling effects that limited detectable short-term improvement. In contrast, participants with more heterogeneous conditions exhibited lower baseline predicted scores, suggesting greater capacity for measurable gains. However, pre- to post-event changes did not differ significantly across diagnosis categories, indicating that the magnitude of acute improvements was broadly similar across diagnostic groups. These findings suggest that baseline satisfaction and ceiling effects, rather than diagnosis alone, may play a key role in shaping detectable acute responses to adaptive sport participation. Interpretation of diagnosis-specific patterns should be made with caution, as the sample size for veterans with sensory impairments was relatively small compared with other diagnosis groups. Additional research with larger and more diagnostically balanced samples is needed to clarify how specific impairment characteristics and baseline psychosocial adaptation influence participation experiences and QoL-related outcomes following adaptive sports participation. Future work should explore activity-specific modifications and complementary outcome measures to better capture meaningful improvements across diverse disability subgroups.

### 4.3 Conceptual models of acute adaptive sports effects on QoL-related outcomes

The findings from this study are consistent with a conceptual model in which acute changes in QoL-related outcomes following participation in adaptive sporting events appear to be shaped by baseline satisfaction with social and leisure participation, as well as broader psychosocial factors related to adaptation and expectations for physical activity. Veterans entered adaptive sporting events with differing baseline levels of satisfaction, partially influenced by service era and medical diagnosis. Veterans from more recent service eras demonstrated lower baseline QoL-related outcomes, a pattern that may reflect differences in expectations for maintaining an active lifestyle or earlier stages of psychosocial adaptation to disability-related changes. In contrast, veterans from earlier service eras exhibited higher baseline satisfaction, potentially reflecting longer-term adaptation and recalibration of expectations over time.

Participation in adaptive sporting events provides a short-term stimulus that includes physical activity, social engagement, peer support and interaction, and opportunities for skill mastery. The acute response to this stimulus appears to be moderated by baseline satisfaction with social and leisure participation rather than by service era alone. Veterans with lower or intermediate baseline PROMIS T-scores demonstrated greater observable improvements following participation, whereas those with higher baseline satisfaction exhibited smaller changes, consistent with ceiling effects inherent to satisfaction-based measures. Within this framework, subgroup differences across service eras may reflect variation in baseline satisfaction and psychosocial context rather than fundamentally different responsiveness to adaptive sports participation. This model suggests that acute improvements in QoL-related outcomes emerge not solely from physical capacity or sport type, but from the alignment between baseline satisfaction, psychosocial readiness, and the inclusive structure of adaptive sporting events.

Importantly, observed improvements in QoL-related outcomes reflect only immediate psychosocial responses to a single-day event and do not permit inference about long-term sustainability. Further, the voluntary nature of participation in the adaptive sporting events may have influenced baseline PROMIS scores, which may partially explain the observed subgroup differences across both service era and medical diagnosis. Baseline differences in motivation, readiness for engagement, and interest in physical activity were not measured in this study and may have contributed to the observed findings. Future research should use standardized measurements to identify these parameters prior to participation in an adaptive sporting event and focus on identifying the long-term impact of these events for veterans of differing service eras and medical diagnoses.

### 4.4 Limitations

Several limitations should be considered when interpreting these findings. First, this study examined acute, short-term changes associated with participation in a single-day event, limiting conclusions about long-term effects. Longitudinal studies with follow-up assessments are needed to determine whether observed improvements are sustained over time. Second, the sample included a substantially higher proportion of male participants, reflecting the broader veteran population, limiting the ability to examine sex-based differences. Future research should aim to recruit more women veterans to support these subgroup analyses. Third, participation in adaptive sporting events was voluntary, raising the possibility of self-selection bias. Veterans who chose to participate may differ in motivation, readiness for engagement, or interest in physical activity compared with those who did not. Such selection effects may have influenced baseline PROMIS scores and observed subgroup differences and should be considered when interpreting these findings. Fourth, the diagnostic categories used in this study were necessarily broad, given the clinical heterogeneity of participants' conditions. Thus, specific diagnoses within a category may show greater QoL improvement than others, and broad conclusions based on medical diagnoses should be interpreted accordingly. Finally, participants were drawn from adaptive sporting events in the New York and New Jersey region, which may limit generalizability to veterans in other geographic areas.

## 5 Conclusions

Participation in single-day community-based adaptive sporting events was associated with acute improvements in QoL-related outcomes among veterans living with disabilities. These findings support adaptive sporting events as a meaningful, low-barrier opportunity for enhancing psychosocial well-being and engagement in active living beyond the traditional clinical setting. Observed differences in overall QoL-related scores across service eras and between some medical diagnostic groups underscore the importance of considering baseline expectations, psychosocial adaptation, and measured context when interpreting short-term responses. However, the reported results are restricted to short-term effects, as participants were surveyed two to four weeks before and then immediately after taking part in a one-day adaptive sporting event. Continued investigation into the mechanisms underlying these benefits may inform the development of inclusive, targeted adaptive sports programs that promote active living, psychosocial well-being, and QoL among veterans living with disabilities.

## Data Availability

The original contributions presented in the study are included in the article/Supplementary Material, further inquiries can be directed to the corresponding author.
