# Supplementary material for: Short-term benefits of adaptive sporting events on social and leisure satisfaction in veterans with disabilities: impact of military service era and medical diagnosis
Source: Front Sports Act Living. 2026 Jun 19;8:1773675. doi: 10.3389/fspor.2026.1773675 (PMC13328358; doi:10.3389/fspor.2026.1773675)
Supplement: Supplementary file 1 [file Table1.docx]

| **Variable** | **Summary** |
| --- | --- |
| Time | Pre-Event  Post-Event |
| Military Service Era | Vietnam (1955-1975)  Post-Vietnam (1975-1990)  Gulf (1990-1991)  Post-Gulf (1992-2000)  OEF/OIF (2001-2021) |
| Medical Diagnosis | Limb Loss (Upper and/or Lower)  Mental Health (e.g., PTSD, anxiety, depression)  Sensory Impairment (e.g., vision or hearing loss)  Musculoskeletal Impairments (e.g., Muscular Dystrophy)  Neurological Impairments (e.g., Spinal Cord Injury) |
| Event Type | Kayaking and Sailing  Cycling and Hiking |
| Sex | Male  Female |
| *Note:* OEF/OIF: Operation Enduring Freedom/Operation Iraqi Freedom; PTSD: Post Traumatic Stress Disorder. | |

**Supplementary Table A.** Summary table of fixed effects variables.
