# Supplementary material for: Short-term benefits of adaptive sporting events on social and leisure satisfaction in veterans with disabilities: impact of military service era and medical diagnosis
Source: Front Sports Act Living. 2026 Jun 19;8:1773675. doi: 10.3389/fspor.2026.1773675 (PMC13328358; doi:10.3389/fspor.2026.1773675)
Supplement: Supplementary file 2 [file Table2.docx]

|  | **Response: T-Scores (Scaled)** | | |
| --- | --- | --- | --- |
| ***Predictors*** | ***Estimates*** | ***Standard Error*** | ***Credible Interval (95%)*** |
| (Intercept) | 1.20 | 0.14 | 0.94 – 1.47 |
| **Time [Post]** | **0.23** | **0.10** | **0.02 – 0.44** |
| **Service Era [Post-Vietnam]** | **-0.17** | **0.10** | -0.37 – 0.02 |
| Service Era [Gulf] | -0.05 | 0.15 | -0.33 – 0.25 |
| **Service Era [Post-Gulf]** | **-0.31** | **0.17** | -0.63 – 0.02 |
| **Service Era [OEF/OIF]** | **-0.36** | **0.12** | **-0.59 – -0.11** |
| **Event [PoH]** | **-0.16** | **0.06** | **-0.29 – -0.04** |
| **Sex [M]** | **0.21** | **0.10** | **0.02 – 0.41** |
| Diagnosis [Limb Loss] | 0.13 | 0.18 | -0.22 – 0.49 |
| Diagnosis [Musculoskeletal] | -0.20 | 0.14 | -0.47 – 0.07 |
| Diagnosis [Neuro] | -0.15 | 0.11 | -0.37 – 0.08 |
| Diagnosis [Sensory] | -0.15 | 0.13 | -0.41 – 0.11 |
| Time [Post] × Diagnosis [Limb Loss] | -0.18 | 0.21 | -0.61 – 0.24 |
| Time [Post] × Diagnosis [Musculoskeletal] | 0.06 | 0.17 | -0.26 – 0.39 |
| Time [Post] × Diagnosis [Neuro] | -0.04 | 0.13 | -0.29 – 0.22 |
| Time [Post] × Diagnosis [Sensory] | -0.16 | 0.14 | -0.45 – 0.12 |
| **Random Effects** |  |  |  |
| σ^2^ | 0.00 |  |  |
| τ_00_ _Individual_ | 0.02 |  |  |
| ICC | 0.07 |  |  |
| N _Individual_ | 120 |  |  |
| Observations | 452 |  |  |
| Marginal R^2^ / Conditional R^2^ | 0.145 / 0.213 |  |  |

**Supplementary Table B.** Predicted T-Scores (Beta Model Component).
