# Supplementary material for: Short-term benefits of adaptive sporting events on social and leisure satisfaction in veterans with disabilities: impact of military service era and medical diagnosis
Source: Front Sports Act Living. 2026 Jun 19;8:1773675. doi: 10.3389/fspor.2026.1773675 (PMC13328358; doi:10.3389/fspor.2026.1773675)
Supplement: Supplementary file 3 [file Table3.docx]

|  | **Response: T-Scores (Scaled)** | | |
| --- | --- | --- | --- |
| ***Predictors*** | ***Estimates*** | ***Standard Error*** | ***Credible Interval (95%)*** |
| ZOI (Intercept) | -0.99 | 0.24 | 2.90 – 3.32 |
| **ZOI Time [Post]** | **0.68** | **0.19** | **0.30 – 1.06** |
| ZOI Service Era [Post-Vietnam] | -0.18 | 0.21 | -0.60 – 0.23 |
| ZOI Service Era [Gulf] | 0.28 | 0.28 | -0.28 – 0.83 |
| ZOI Service Era [Post-Gulf] | 0.22 | 0.30 | -0.40 – 0.82 |
| **ZOI Service Era [OEF/OIF]** | **-0.50** | **0.26** | **-1.01 – -0.01** |
| **ZOI Diagnosis [Limb Loss]** | **0.59** | **0.27** | **0.07 – 1.11** |
| **ZOI Diagnosis [Musculoskeletal]** | **0.56** | **0.24** | **0.09 – 1.04** |
| **ZOI Diagnosis [Neuro]** | -0.39 | 0.22 | -0.83 – 0.04 |
| **ZOI Diagnosis [Sensory]** | **-0.82** | **0.26** | **-1.34 – -0.31** |
| Observations | 452 | | |

**Supplementary Table C.** Zero-One-Inflated (ZOI) Model Component
