# Supplementary material for: Short-term benefits of adaptive sporting events on social and leisure satisfaction in veterans with disabilities: impact of military service era and medical diagnosis
Source: Front Sports Act Living. 2026 Jun 19;8:1773675. doi: 10.3389/fspor.2026.1773675 (PMC13328358; doi:10.3389/fspor.2026.1773675)
Supplement: Supplementary file 4 [file Table4.docx]

**Supplementary Table D.** Contrasts of Predicted T-Scores.

| **Comparison** | **Predicted T-score** | **Estimate** | **Lower 95% Credible Interval** | | **Upper 95% Credible Interval** |
| --- | --- | --- | --- | --- | --- |
| **Military Service Era** | | | | | |
| *OEF/OIF* | 52.63 |  |  | |  |
| Vietnam |  | **-4.70** | **-7.44** | | **-1.96** |
| Post-Vietnam |  | **-2.74** | **-5.55** | | **-0.01** |
| Gulf |  | **-5.22** | **-8.55** | | **-1.84** |
| Post-Gulf |  | -2.94 | -6.94 | | 1.22 |
| *Post-Gulf* | 55.59 |  |  | |  |
| Vietnam |  | -1.70 | -5.33 | | 1.61 |
| Post-Vietnam |  | 0.24 | -3.64 | | 3.79 |
| Gulf |  | -2.28 | -6.47 | | 1.91 |
| *Gulf* | 57.86 |  |  | |  |
| Vietnam |  | 0.56 | -2.27 | | 3.14 |
| Post-Vietnam |  | 2.52 | -0.52 | | 5.28 |
| *Post-Vietnam* | 55.33 |  |  | |  |
| Vietnam |  | -1.96 | -3.96 | | 0.06 |
| *Vietnam* | 57.31 |  |  | |  |
| **Medical Diagnosis** | | | | | |
| *Sensory* | 52.59 |  |  |  | |
| Neuro |  | -1.75 | -4.46 | 0.90 | |
| Musculoskeletal |  | **-5.05** | **-8.10** | **-1.90** | |
| Limb Loss |  | **-6.69** | **-9.80** | **-3.32** | |
| Mental Health |  | **-4.35** | **-6.97** | **-1.75** | |
| *Neuro* | 54.35 |  |  |  | |
| Musculoskeletal |  | **-3.29** | **-5.89** | **-0.49** | |
| Limb Loss |  | **-4.92** | **-7.79** | **-1.66** | |
| Mental Health |  | **-2.60** | **-4.74** | **-0.43** | |
| *Musculoskeletal* | 57.64 |  |  |  | |
| Limb Loss |  | -1.63 | -4.88 | 1.83 | |
| Mental Health |  | 0.69 | -1.86 | 3.15 | |
| *Limb Loss* | 59.28 |  |  |  | |
| Mental Health |  | 2.33 | -0.68 | 4.94 | |
| *Mental Health* | 56.96 |  |  |  | |
| **Event Type** | | | | | |
| Pedals of Honor - Heroes on the Hudson |  | **-1.43** | **-2.49** | **-0.34** | |
