# Supplementary material for: Short-term benefits of adaptive sporting events on social and leisure satisfaction in veterans with disabilities: impact of military service era and medical diagnosis
Source: Front Sports Act Living. 2026 Jun 19;8:1773675. doi: 10.3389/fspor.2026.1773675 (PMC13328358; doi:10.3389/fspor.2026.1773675)
Supplement: Supplementary file 5 [file Table5.docx]

| **Medical Diagnosis** | **Time** | **Estimate** |
| --- | --- | --- |
| Mental Health (n=30) | Pre | 55.00 |
|  | Post | 58.92 |
| Limb Loss (n=10) | Pre | 57.99 |
|  | Post | 60.64 |
| Musculoskeletal (n=20) | Pre | 55.28 |
|  | Post | 60.06 |
| Neurologic (n=38) | Pre | 52.42 |
|  | Post | 56.28 |
| Sensory (n=22) | Pre | 51.40 |
|  | Post | 53.80 |
| **Military Service Era** | **Time** | **Estimate** |
| Vietnam (n=35) | Pre | 55.65 |
|  | Post | 58.98 |
| Post Vietnam (n=48) | Pre | 53.51 |
|  | Post | 57.19 |
| Gulf (n=11) | Pre | 56.14 |
|  | Post | 59.59 |
| Post Gulf (n=10) | Pre | 53.54 |
|  | Post | 57.64 |
| OEF/OIF (n=20) | Pre | 51.65 |
|  | Post | 55.47 |

**Supplementary Table E.** Pre-Post T-scores for subgroups of Medical Diagnosis and Military Service Era.
