# Supplementary figures and images for: Short-term benefits of adaptive sporting events on social and leisure satisfaction in veterans with disabilities: impact of military service era and medical diagnosis
Source: Front Sports Act Living. 2026 Jun 19;8:1773675. doi: 10.3389/fspor.2026.1773675 (PMC13328358; doi:10.3389/fspor.2026.1773675)

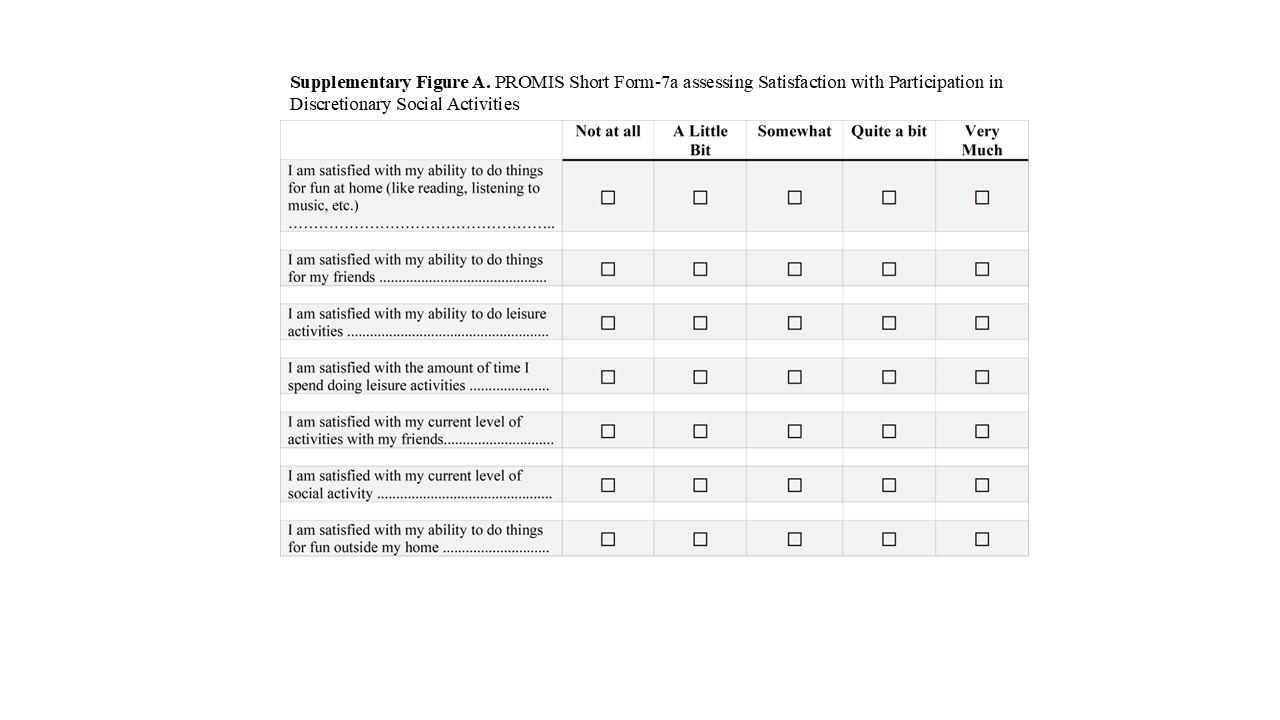

Supplement: Supplementary file 6 [file Image1.tif]
